# Survey on cervical cancer knowledge and its influencing factors among 2,578 women in Shache county, Kashi, China

**DOI:** 10.1186/s12905-023-02390-4

**Published:** 2023-05-09

**Authors:** Yilidana Mijiti, Hainiguli Yusupu, Haixia Liu, Xuefeng Zhang, Gulikezi Maimaiti, Reyilaimu Kawuli, Cailing Ma

**Affiliations:** 1 Department of Gynecology, State Key Laboratory of Pathogenesis, Prevention and Treatment of High Incidence Diseases in Central Asia, Xinjiang Key Laboratory of Medical Animal Model Research, The First Affiliated Hospital of Xinjiang Medical University, Urumqi, Xinjiang P.R. China; 2 Department of Gynecology, Shache county people’s hospital, Kashi, Xinjiang P.R. China

**Keywords:** Cervical cancer, Influencing factor, Pap test, Prevention

## Abstract

**Background:**

In the southern part of Xinjiang, the incidence and mortality rates of the cervical cancer among Uyghurs are significantly higher than those of other ethnic groups living in the same environment, and their mortality rate takes the first place among ethnic minorities in China.

**Objective:**

To assess the level of cervical cancer knowledge by Questionnaire survey using the scoring system and its influencing factors among women in Shache county, Kashi, China.

**Method:**

Based on the cervical cancer health education carried out by the medical team of the county hospital to the residents in the urban and rural areas of Shache County from September 1st, 2022, to September 30th, 2022, a cluster sampling was conducted at the same time and a total of 2578 women were included. The questionnaire survey includes resident basic information, and their knowledge about cervical cancer which was evaluated by the scoring system. The scoring system of the knowledge about cervical cancer includes 4 items, the total score was 8 point and scored more than 4 points was used as knowledge knowing, the knowledge of cervical cancer (unknown = 0; known = 1) was used as the dependent variable. Six factors include residence area (urban or rural), age, ethnic group, educational level, occupation, and the ways to acquire knowledge access were used as independent variables. The retrieved questionnaire was entered by the medical staff, and the Excel software was used for duplicate verification. Chi-square test and unconditional logistic regression analysis were used for statistical analysis.

**Results:**

Of the 2578 study subjects, 1591 were from rural areas and 987 were from the urban areas, and the differences were statistically significant (P < 0.001). Based on the scoring system of the knowledge about cervical cancer, the knowledge knowing accounts for only 34.1%. Multivariate logistic regression analysis showed that living in the urban(AOR = 1.358,95% CI:1.111–1.659), occupation of non-farming and non-housewife(AOR = 2.680,95%CI:2.126–3.377), education level of high school and above(AOR = 1.388,95%CI:1.125–1.712), and four or more access to knowledge(AOR = 1.446,95%CI:1.099–1.903) were protective factors for cervical cancer knowledge knowing.

**Conclusion:**

Based on the questionnaire survey, the level of cervical cancer knowledge among women in Shache county was inadequate. Considering above mention influencing factors, it is necessary for the medical and health institutions to take various measures to carry out targeted health education on cervical knowledge for women in Shache county.

**Supplementary Information:**

The online version contains supplementary material available at 10.1186/s12905-023-02390-4.

## Introduction

Cervical cancer is the second most common malignant tumor in women and the most prevalent malignant tumor in the female reproductive system. About 85% of cervical cancer cases occur in less developed countries compared to developed countries [1]. China is one of the high incidence areas of cervical cancer [2]. Especially in the central and western regions, it is highly prevalent and poses a serious threat to the health of women in China. Compared to developed countries, cervical cancer screening rates in China are still low, especially in rural areas [3]. This regional variation can be explained by the different levels of cervical cancer screening knowledge [4]. A study report found [5] that the degree of women’s knowledge about cervical cancer prevention and control affects the development of cervical cancer prevention and treatment to some extent. Improving women’s knowledge level is clinically important for early detection, early diagnosis, and treatment of cervical disease [6]. Therefore, it is particularly important to understand the relevant factors affecting cervical cancer screening.

Xinjiang, located in northwestern China, is one of the high incidence areas of cervical cancer in China. Uyghurs are one of the major ethnic groups in Xinjiang, especially in the southern part of Xinjiang, and their cervical cancer incidence and mortality rates are significantly higher than those of other ethnic groups living in the same environment, and their mortality rate takes the first place among ethnic minorities in China [7], and most cervical cancer cases, once detected, are in the middle and late stages, among which who suffered cervical cancer have never undergone cervical cancer screening or even routine gynecological examination. Shache County, which belongs to Kashgar region of Xinjiang Uighur Autonomous Region, and is a large county with a large population mainly in agriculture. In this study, Shache County was taken as a study subject and the investigation was conducts about the level of cervical cancer screening and protection knowledge among different groups women in rural and urban areas by questionnaires, and the influencing factors of their knowledge acquisition were analyzed. The difference of the present study from previous studies was the comparative analysis between rural and urban areas, which provided scientific basis for more effective and targeted cervical cancer prevention and treatment in local and different groups in the future.

## Object and method

**Research object** This study was based on the cervical cancer health education of residents in the county seat, rural communities and village committees of Shache County conducted by the medical team of the county Hospital from September 1st 2022 to September 30th 2022. Convenient sampling was carried out in rural townships and urban communities, and 7 townships and 22 communities participated in the study. The women who participated in the questionnaire were all permanent residents. The inclusion criteria of the research objects were women aged 15–70 who had sex, and those who suffered from serious mental disorders or behavioral and intellectual disabilities, had difficulty in understanding the content of the questionnaire and could not cooperate with the completion of the questionnaire were excluded.

**Questionnaire** Through a review of domestic and international literature [8–13] and expert consultation, the “Cervical Cancer Prevention and Treatment Awareness Questionnaire for Women in Shache County” was designed and distributed to those who met the criteria within their jurisdiction through the county women’s committee, maternal and child health center, community health institutions, village committees, community cadres, and cadres of each village at different levels. To ensure that there is no language misunderstanding among the surveyed women, the questionnaire is bilingual in Chinese and Uyghur. The questionnaire covers the basic information of the study subjects, such as age, marital status, education level, occupation, knowledge about cervical cancer disease itself and its influencing factors, preventive measures, etc. The way to get information about cervical cancer-related knowledge, the way to get knowledge about cervical cancer prevention and treatment. The survey focused on the reasons for not having cervical cancer screening. For illiterate women, the questionnaire was conducted by verbal translation under the help from their family member which was also supervised by trained professional team members to ensure the fully understanding and accuracy of the questions. The questionnaires were distributed and collected by the medical officers of the medical teams in each jurisdiction, and the questionnaires were collected after completion of on-site examination.

### Statistical analysis

The recovered questionnaires were double-entered and verified using Excel software. The statistical software SPSS27.0 was used for statistical analysis, t-test was used for descriptive analysis, chi-square test was used for comparison of rates between groups, P < 0.05 was considered statistically significant, and logistic regression analysis was used for the analysis of factors influencing the degree of cervical cancer awareness.

## Results

### Basic information

In this study, a total of 2578 respondents completed the questionnaire survey, 987 cases (38.29%) in the county area, and 1591 cases (61.71%) in rural area. The final included subjects were aged 15 to 67 years old, with an average of 32 ± 1 years, of which 1178 people aged ≤ 30 years old (45.7%), 1124 people aged 31 to 44 years old (43.6%), 276 people aged ≥ 45 years old (0.7%). The marital status includes 1964 married people (76.18%), 261 unmarried people (10.12%), and 353 others (13.7%) such as divorced or widowed. There were 93.5% of Uyghur, 5.0% of Han and 1.5% of other ethnic group. The education level was divided into three groups: junior high school/junior college and below, high school/college, and bachelor’s degree and above, which accounts for 54.9%, 36.5%, and 8.6%, respectively. Occupation includes enterprise 55.0%, farming 22.9%, housewife 18.1%, and individual 4.1%. The difference between the socio-demographic status of women in the urban areas and rural areas was statistically significant. The basic conditions of the survey respondents are shown in Table 1.

**Table 1:** Comparison of socio-demographic status of women in urban and rural areas (n%)

Features	Urban	Rural	Total	X2	P
**Ethnicity**				138.5	< 0.01
Uyghur	853 (86.4%)	1557 (97.9%)	2410 (93.5%)		
Han-Chinese	111 (11.2%)	19 (1.2%)	130 (5.0%)		
Other races	23 (2.3%)	15 (0.9%)	38 (1.5%)		
**Age**				151.2	< 0.01
≤30	345 (35.0%)	833 (52.4%)	1178 (45.7%)		
31–44 years old	452 (45.8%)	672 (42.2%)	1124 (43.6%)		
≥45	190 (19.3%)	86 (5.40%)	276 (10.7%)		
**Education level**				561.9	< 0.01
≤ Junior High School / College	271 (27.5%)	1145 (72.0%)	1416 (54.9%)		
High school/college	523 (53.0%)	417 (26.2%)	940 (36.5%)		
≥ Bachelor	193 (19.5%)	29 (1.8%)	222 (8.6%)		
**Career Status**				602.1	< 0.01
Farming	42 (4.3%)	547 (34.4%)	589 (22.9%)		
Homemakers	77 (7.8%)	389 (24.5%)	466 (18.1%)		
Enterprises and Businesses	839 (85.0%)	579 (36.4%)	1418 (55.0%)		
Individuals	29 (2.9%)	76 (4.8%)	105 (4.1%)		
**Contraceptive methods**				124.2	< 0.01
IUD ring	593 (6.1%)	1233 (77.5%)	1826 (70.8%)		
Condoms	127 (12.9%)	59 (3.7%)	186 (7.2%)		
No measures	172 (17.4%)	167 (10.5%)	339 (13.2%)		
Contraceptives	31 (3.1%)	28 (1.8%)	59 (2.3%)		
Safety Period	64 (6.5%)	104 (6.5%)	168 (6.5%)		

* **IUD** ring, intrauterine device

### Knowledge about cervical cancer

The cervical cancer screening rate in this surveyed population was 9.6%, among which 58.7% of the screened women lived in the urban area. Regarding to the awareness of the cervical cancer was related to HPV infection, the rate of awareness was 68.6%, among which 57.3% of them live in rural area. Regarding the HPV vaccination, the overall vaccination rate was 5.1%, among which 54.5% who lived in the urban area. The rate of awareness on the cervical cancer prevention by early screening accounts for 85.5%, and the difference in knowledge about cervical cancer between urban and rural areas was statistically significant. The details are shown in Table 2.

**Table 2:** Knowledge about cervical cancer among urban and rural women [n%]

Knowledge about cervical cancer	Urban	Rural	Total	X2	P
**Did you know about cervical cancer screening**				116.9	< 0.001
Heard of but not screened	352 (35.7%)	720 (45.2%)	1072 (41.6%)		
Never heard of it	121 (12.3%)	356 (22.4%)	477 (18.5%)		
Screened (≥ 1 time)	145 (14.7%)	102 (6.4%)	247 (9.6%)		
Understood but not screened	369 (37.3%)	413 (25.0%)	782 (30.3%)		
**Is it known that cervical cancer is associated with HPV infection**				47.3	< 0.001
Know	756 (76.6%)	101 (63.7%)	1769 (68.6%)		
No idea	231 (23.4%)	578 (36.3%)	809 (31.4%)		
**Have you heard of the HPV vaccine?**				137.2	< 0.001
No idea	156 (15.8%)	587 (36.9%)	743 (28.8%)		
Injected	72 (7.3%)	60 (3.8%)	132 (5.1%)		
Known but not injected	759 (76.9%)	944 (59.3%)	1703 (66.1%)		
**Did you know that cervical cancer can be detected and prevented early through early screening?**				6.15	0.013
No idea	140 (14.2%)	285 (17.9%)	425 (16.5%)		
Know	847 (85.8%)	1306 (82.1%)	2153 (83.5%)		

*HPV, human papilloma virus

### Factors influencing the knowledge score of cervical cancer

The scoring system of the knowledge about prevention and treatment of cervical cancer includes 4 items: (1) Do you know about cervical cancer screening? (2) Is it known that cervical cancer is associated with HPV infection? (3) Have you heard of the HPV vaccine? (4) Did you know that cervical cancer can be detected and prevented early through early screening? (Specific questions were listed in the Table 3). The total score was 8 point and scored more than 4 points was used as the knowledge knowing. The results showed that age, place of residence, occupation, education level, and access to knowledge were all independent factors influencing knowledge of cervical cancer prevention and treatment, among which living in the urban area, occupation as non-farming or housewife, education level of high school or above, and four or more access to knowledge were protective factors for knowledge of cervical cancer (P < 0.05), as shown in Table 4, and the assignment table is shown in Table 3.

**Table 3:** Cervical cancer-related knowledge assignment

Knowledge about cervical cancer	Assignment
**Do you know about cervical cancer screening**	**3**
Heard of but not screened	0
Never heard of it	0
Screened (≥ 1 time)	2
Understood but not screened	1
**Is it known that cervical cancer is associated with HPV infection**	**1**
Know	1
No idea	0
**Have you heard of the HPV vaccine?**	**3**
Known but not injected	1
No idea	0
Injected	2
**Did you know that cervical cancer can be detected and prevented early through early screening?**	**1**
No idea	0
Know	1

*HPV, human papilloma virus

**Table 4:** Multi-factor logistic regression analysis of women’s cervical cancer knowledge scores

Factors	Comparison Group	B	S.E	Wold x2	P	OR	95% *CI*
Races	Non-native Chinese speakers	0.289	0.185	2.439	0.118	1.335	0.929–1.917
Age	>45 years old	− 0.446	0.145	9.480	0.002	0.640	0.482–0.850
Address	Rural	0.306	0.102	8.933	0.003	1.358	1.111–1.659
Career	Farming + Homemakers	0.986	0.118	69.792	0.000	2.680	2.126–3.377
Education level	≤junior high school/junior college	0.328	0.107	9.382	0.002	1.388	1.125–1.712
Access to knowledge	Less than 4 kinds	0.369	0.140	6.942	0.008	1.446	1.099–1.903

* B, regression coefficients; **SE**, standard error; **P**, p-value; **OR**, odds ratio; **CI**, confidence interval

### Reasons for not performing cervical cancer screening

Among the respondents, 247 of them had cervical cancer screening once or more, while 2331 cases (90.4%) were not screened, and the reasons for further investigation were: no symptoms, no need; what is that? (equal to not knowing about cervical cancer screening at all); fear or shyness of gynecological examination; distrust of doctors (fear of spending money); lack of time to go to the hospital; husband’s lack of support, no accompany; inconvenient transportation; fear of privacy disclosure; economic problems like expensive screening and other nine reasons. Among them, 67.7% of them thought there were no symptoms and no need, ranking first, followed by fear or shyness of performing gynecological examination, ranking second (15.6%). The differences in the situation of reasons for not having cervical cancer screening between county town and rural areas were statistically significant, as shown in Table 5.

**Table 5:** Reasons for not having cervical cancer screening among urban and rural women [n%]

Reasons for not getting screened for cervical cancer	Urban	Rural	Total	X2	P
No symptoms, no need	534 (63.4%)	1045 (70.2%)	1579 (67.7%)	160.8	< 0.001
What’s that?	55 (6.5%)	139 (9.3%)	194 (8.3%)		
Fear or shyness of having a gynecological examination	173 (20.5%)	190 (12.8%)	363 (15.6%)		
I don’t trust the doctor, and I’m afraid I’ll be paid in vain.	12 (1.4%)	10 (0.7%)	22 (0.9%)		
No time to go to the hospital	191 (2.3%)	98 (6.6%)	289 (12.4%)		
Unsupportive husband, unaccompanied	12 (1.4%)	27 (1.8%)	39 (1.7%)		
Poor transportation and inconvenience in getting back and forth	13 (1.5%)	47 (3.2%)	60 (2.6%)		
Fear of privacy breach	26 (3.1%)	71 (4.8%)	97 (4.2%)		
Screening is expensive and economically problematic	29 (3.4%)	103 (6.9%)	132 (5.7%)		

### Access to knowledge on cervical cancer prevention and treatment

**Fig. 1:**
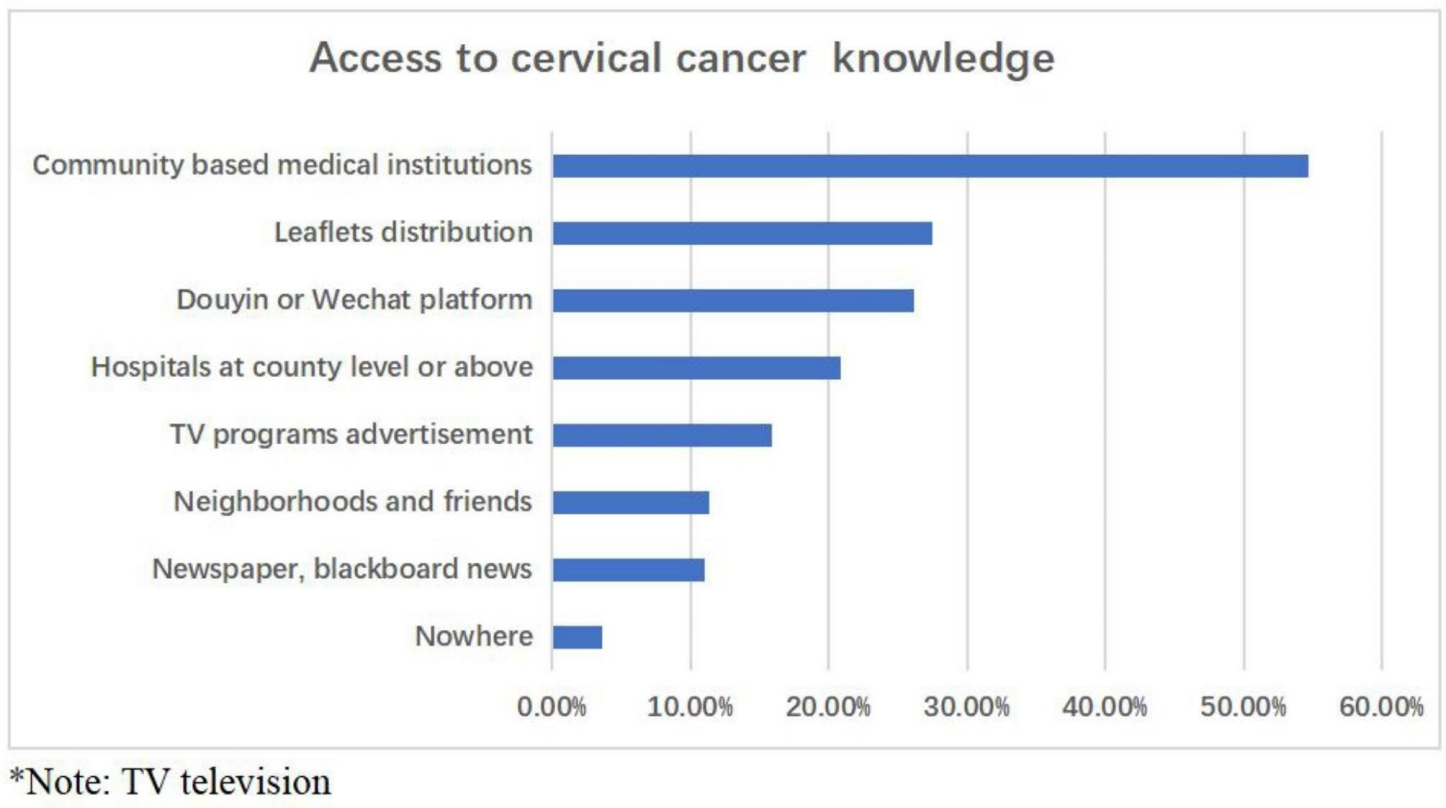
Access to cervical cancer knowledge

Among the 2,578 women, there were 8 ways to accessing the knowledge about cervical cancer, which includes community based medical institutions (54.7%), leaflets distribution (27.5%), Douyin or Wechat platform (26.2%), hospitals at county level or above (20.9%), TV programs advertisement (15.9%), neighbors and friends (11.3%), and newspaper and blackboards news (11.0%). The highest access was through community based medical institutions which include community health prevention institutions or medical institutions. The difference between the different ways of acquiring knowledge was statistically significant between the urban and rural areas (X^2^ = 231.6, p < 0.001), and its distribution is shown in Fig. 1.

## Discussion

Southern Xinjiang, especially in rural areas, with more Uyghur farmers, under the influence of relatively low level of education and economy, early marriage age of women, more frequent deliveries, and relatively poor sanitary conditions, has become a high incidence area for cervical cancer [14]. In the present study, a total of 2578 women lived in Shache county received a questionnaire survey on the level of cervical cancer screening and protection knowledge, the preliminary results showed that living area, education level, occupation, the number of accessing knowledges were significantly associated with the awareness of knowledges related to the cervical cancer screening and protection.

Mei Zhang found in her study that the coverage rate of cervical cancer screening among women aged ≥ 21 years was only 23.6%, which was even lower in resource-poor areas [15], which may be related to the lower awareness and acceptance of cervical cancer screening among women in resource-poor areas and the lower awareness of the population to initiate screening [16], and the lower level of public awareness of cervical cancer screening is also an important reason affecting the coverage rate of cervical cancer screening in China. Only 9.6% of the women in this survey had been screened for cervical cancer, and the screening rate was even lower, indicating that the awareness of screening was weaker among women in this region. 18.5% of women did not know what cervical cancer screening was. The reasons for not screening were analyzed among the 2331 women who did not undergo screening, 67.7% of them thought there was no need for screening because they did not have any symptoms, followed by 15.6% who were afraid or shy to undergo gynecological examination, only 5.7%, 4.2% and 2.6% were considered for expenses, privacy, and traffic problems, respectively. This shows that the acceptance of cervical cancer screening among women in this region is low, and the influence of subjective factors is higher than that of objective factors.

Studies have shown that the level of cervical cancer awareness is a key determinant of women’s participation in cervical cancer screening and that lack of knowledge about cervical cancer screening is a major barrier to women’s participation in cervical cancer screening [17, 18]. In the survey of cervical cancer-related knowledge among the study participants, it was found that there was a significant difference in the awareness rate between rural and county town women, 18.5% of women had never heard of cervical cancer screening, 31.4% of women did not know that cervical cancer was related to HPV infection, only 5.1% of women had received the cervical cancer vaccine, 28.8% of women did not know about the cervical cancer vaccine, and among them 79.0% (587/ 743) of women live in rural areas, which is a big difference from 21.0% in the county (156/743), indicating that the knowledge of cervical cancer in the county is significantly better than that of women living in rural areas, which may be related to the fact that there are more medical institutions in the county town, fewer housewives and farming residents, more opportunities to contact new information, relatively good living conditions, relatively high education level, and relatively strong acceptance of community medical work. In contrast, rural areas are mainly farming, spending most of their time in the fields or at home, with relatively closed information, low literacy, and poor understanding and acceptance ability. Even though most women lack knowledge of cervical cancer protection, 83.5% of women know and believe that cervical cancer can be prevented through early screening, but most of them have a fluke mentality and believe that it is asymptomatic and unnecessary, as shown in the study results. The knowledge of cervical cancer prevention and treatment was assigned a score out of 8, and those who scored ≥ 4 was qualified, accounting for only 34.1% (880/2578), while the remaining 65.9% were not qualified. This indicates a lack of knowledge about cervical cancer in the region, which is consistent with the results of other studies at home and abroad [19–23]. Similar to other studies related to factors influencing knowledge of cervical cancer prevention and treatment [24, 25], knowledge of cervical cancer prevention and treatment in this study was related to age, place of residence, occupation, education level, and access to knowledge, and living in the county, occupation of non-farming and non-housewife, education level of high school and above, and four or more access to knowledge were protective factors for knowledge of cervical cancer. Therefore, in the future, in the work of cervical cancer prevention and treatment, we should make efforts to improve the literacy of residents, do more publicity and lectures, mainly focus on female farmers and housewives, improve the literacy of these women, so as to improve the overall knowledge of cervical cancer prevention and treatment and further increase the screening rate, because the level of education is one of the main factors to determine women’s awareness of cervical cancer and women’s participation in regular screening [26].

The results of several studies [20, 21] point out that the lack of health awareness and effective ways to obtain knowledge, the knowledge related to cervical cancer is even weaker, about 3.6% of women in this study did not obtain any knowledge of cervical cancer. Song Bo and Li Jianmei [11, 22] also pointed out in her study that the more channels rural women have to receive knowledge about cervical cancer prevention and treatment, the higher the degree of knowledge acquisition, and the stronger the initiative to participate in cervical cancer screening. Among the survey respondents, there were 8 channels of knowledge acquisition, and only 9.9% (254/2578) had more than 4 channels of acquisition, among which the most frequent sources of acquisition were community health prevention institutions or medical institutions, followed by community-issued leaflets, then county-level hospitals and above, and the lowest were newspapers and blackboards. National and international studies have shown [27, 28] that regular visits to physicians can awake and reinforce women’s health awareness and motivate them to learn about cervical cancer screening initiatively. In addition, the study still found that women who received lectures from professionals scored much higher than those who received them through other means, which is consistent with the Bo Song’s study [11]. This indicates that the degree of knowledge acquisition varies greatly depending on the means of knowledge acquisition. Therefore, to strengthen the knowledge of cervical cancer protection and increase the early screening rate of women for cervical cancer in the future, the publicity and education work of professional institutions such as communities, community health prevention institutions and hospitals need to be further strengthened, and the awareness rate should be further increased by strengthening the dedicated education in medical institutions, TV media, brochures, and publicity boards.

The limitation of this study is that there are few questions about cervical cancer prevention and treatment in the questionary, which may lead to deviation bias in the composition of knowledge. Another limitation of the present study is that the regular resident is defined as temporal cut-off of six month, which lead to possible collection bias. In future research, study subject should be selected as regular resident who lived in urban or rural area more than 12 month, and we should add some detailed questions about the symptoms of cervical cancer, risk factors of disease, and preventive measures, to achieve a more appropriate investigation.

## Conclusion

The level of cervical cancer screening and protection knowledge among women in Shache county was inadequate, which need to be promoted by regular communication between healthcare professionals and women lived in rural area, strengthen their health education, and establish a complete health screening system, which could potentially increase the early screening rate of cervical cancer, and reduce the late incidence of cervical cancer.

## Electronic supplementary material

Below is the link to the electronic supplementary material.

Supplementary Material 1: Cervical cancer prevention and treatment awareness questionnaire for women in Shache County

## Data Availability

All data generated or analyzed during this study are included in this published article.
