# Supplementary material for: Survey on cervical cancer knowledge and its influencing factors among 2,578 women in Shache county, Kashi, China
Source: BMC Womens Health. 2023 May 9;23:246. doi: 10.1186/s12905-023-02390-4 (PMC10170853; doi:10.1186/s12905-023-02390-4)
Supplement: Supplementary file 1 — Supplementary Material 1: Cervical cancer prevention and treatment awareness questionnaire for women in Shache County [file 12905_2023_2390_MOESM1_ESM.pdf]

Cervical Cancer Prevention and Treatment Awareness Questionnaire for Women in Shache County

For better understand the cognition of women on cervical cancer screening in Shache County, improve the early screening rate of cervical cancer screening, improve the early detection rate, and reduce the fatality rate, the following questionnaire survey was conducted.

1.Your ethnic group

①The Uygur
②Han Chinese
③Tajik
④Khalkhas
⑤the Hui
⑥Kazak
⑦Other ethnic groups

2.Your age？ （ ）

3.Your marital status？

①Married
②Single
③Divorced
④Unmarried but have sexual life

4. Your residential address?

①Shache urban area, the specific address（ ）
②Shache rural area，the specific address（ ）

5. Your occupation?

① Farmer
② Housewife
③ Enterprise units
④ Teacher
⑤ Medical staff
⑥ Individual
⑦ Other public institutions

6. Your degree of education？

① Illiteracy
② Primary school
③ Junior high school or technical secondary school
④ High school or junior college
⑤ Bachelor or above degree

7. **Did you know about cervical cancer screening**?
①Heard of but not screened
②Never heard
③Screened (≥1 time)
④Understood but not screened

8、**Do you know that cervical cancer is associated with HPV infection?**
① Yes，I do
② No，I don't

9. **Have you heard of the HPV vaccine?**
①No idea
②Injected
③Known but not injected

10、If you do not perform cervical cancer screening, please explain why (multiple option)
①No symptoms, no need
②Screening is expensive and economically problematic
③No time to go to the hospital
④Fear or shyness of having a gynecological examination
⑤Fear of privacy
⑥Inconvenience of transportation
⑦I don't trust the doctor, and I'm afraid I'll be paid in vain.
⑧What's that?
⑨Unsupportive husband, unaccompanied

11、Did you know that cervical cancer can be detected and prevented early through early screening?
①Yes，I do
②No，I don't

12. Where do you learn about cervical cancer and HPV from (multiple option)
① Neighbors and friends
② TV programs
③ Douyin platform
④ From leaflets distributed in the community
⑤ From community health prevention institutions or medical institutions,
⑥ From hospitals at county level or above
⑦ Never heard
⑧ From other ways like newspaper, blackboard news
⑨ WeChat platform

13、Your way of contraception

①Intrauterine device
②Condom
③No contraceptive measures
④Safe period contraception
⑤Contraceptive pill

14、The platform way for you to obtain new information is (multiple option)
① TV program
② Newspaper
③ Wechat
④ Douyin
⑤ Other medias
⑥ Community education
